# Dry Glass Reference Perturbation Theory Predictions of the Temperature and Pressure Dependent Separations of Complex Liquid Mixtures Using SBAD-1 Glassy Polymer Membranes

**DOI:** 10.3390/membranes12070705

**Published:** 2022-07-12

**Authors:** Bennett D. Marshall, Wenjun Li, Ryan P. Lively

**Affiliations:** 1 ExxonMobil Technology and Engineering Company, Annandale, NJ 08801, USA; wenjun.li@exxonmobil.com; 2 School of Chemical and Biomolecular Engineering, Georgia Institute of Technology, Atlanta, GA 30332, USA; ryan.lively@chbe.gatech.edu

**Keywords:** glassy polymer, membranes, theory, petrochemical

## Abstract

In this work we apply dry glass reference perturbation theory (DGRPT) within the context of fully mutualized diffusion theory to predict the temperature and pressure dependent separations of complex liquid mixtures using SBAD-1 glassy polymer membranes. We demonstrate that the approach allows for the prediction of the membrane-based separation of complex liquid mixtures over a wide range of temperature and pressure, using only single-component vapor sorption isotherms measured at 25 °C to parameterize the model. The model was then applied to predict the membrane separation of a light shale crude using a structure oriented lumping (SOL) based compositional model of petroleum. It was shown that when DGRPT is applied based on SOL compositions, the combined model allows for the accurate prediction of separation performance based on the trend of both molecular weight and molecular class.

## 1. Introduction

The separation of complex liquid mixtures by distillation is energy intensive due to the necessity of phase change. Membranes provide a low energy alternative to distillation by negating the requirement of phase change, which may help decarbonize the petrochemical industry via the debottlenecking of existing and proposed distillation columns [1,2]. For membranes to become widely adopted in the industry, there is a need to develop modeling platforms that allow for the prediction of separations of complex liquid mixtures over a wide range of temperature and pressure.

Glassy polymer membranes are beginning to be used in hydrocarbon separations [3]. Glassy polymers are polymers at a temperature less than their glass transition temperature. In this state, the polymer chains are arrested in an out of equilibrium configuration. Based on extensive analysis in the gas separation literature [4,5,6], the membrane microstructure is often imagined as a transiently porous structure in which molecules sorb from the feed and diffuse through the porous membrane via transient-free volume elements that open and close via random thermal fluctuations of the glassy polymer chains. The selectivity between two species can be estimated as the product of their guest diffusivity ratio and solubility ratio in the membrane.

The microstructure and chain dynamics of the polymer membrane are very different in the case of organic liquid separations such as pervaporation and organic solvent reverse osmosis. Here, the membrane dilates due to the high solubility of common organic liquids in most glassy polymers. The organic liquids often induce plasticization of the polymer glasses, thus enhancing chain mobility. In situations with strong solvent–polymer interactions, these two phenomena can couple to result in the formation of continuum-like clusters of molecules permeating and diffusing through the polymer membrane. It is worth contrasting this against the case of gas membrane separation, in which the gases are often sufficiently dilute in the glassy polymer system such that the gas diffusivity can be calculated via activation rates of single polymer segment motions and individual gas molecule diffusive jumps.

For polymers containing aromatic backbones such as Matrimid, PIM-1, and SBAD-1, aromatic hydrocarbons swell the polymer to a much higher degree than saturated hydrocarbons [7]. In extreme cases, the solvent-swollen polymer can behave more like a liquid phase rather than a glassy polymer. Hence, the character of the membrane, glass-like or liquid-like, really depends on the sorbed liquid composition. 

**Figure 1:**
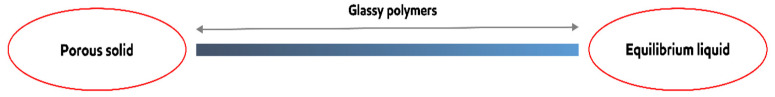
Continuum behavior of a glassy polymer membrane.

Therefore, guest transport behaviors through the same glassy polymer membrane can span the range from individual molecular motions (e.g., light gas permeation) to the continuum-level convection of fluids through a dilated polymer such that the solvent–polymer system behaves as an equilibrium liquid polymer (Figure 1).

**Figure 2:**
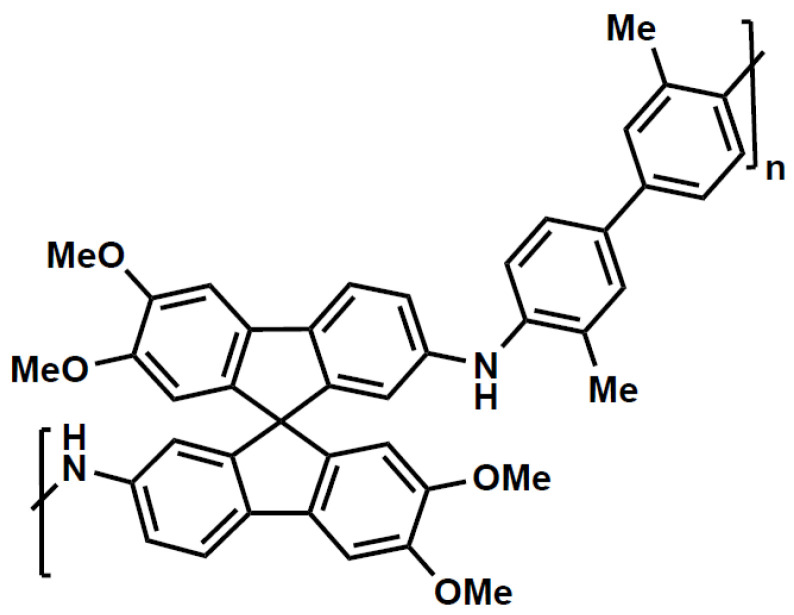
Diagram of SBAD-1 repeat unit [8].

In this work we restrict our attention to the glassy polymer SBAD-1 [3], which is a spirocyclic polymer with N-aryl bonds (Figure 2). This membrane was chosen because there has been an extensive set of pure component sorption and diffusion measurements as well as an extensive set of membrane separations of liquid hydrocarbons using this polymer.

Mathias et al. [8] measured the pure component vapor solubility as well as hydraulic permeability of nine hydrocarbon species (Table 1) in SBAD-1, while Thompson et al. [3] measured the temperature and pressure dependence of the separation of a liquid mixture composed of these nine species. In addition, the membrane separation of a light shale crude oil was also reported [3].

In a recent publication, Marshall et al. [9] applied the Maxwell-Stefan [10] diffusion equation to the pure hydraulic liquid permeation data of Mathias et al. [8]. The Maxwell–Stefan equation was applied in the mole fraction representation so that the extracted diffusivities have a clear molecular interpretation. For a pure species *i* diffusing through an immobile polymer membrane along the dimension *z*, the Maxwell–Stefan equation simplifies to,

(1) $$N_{i}=-\frac{\rho_{i}}{k_{B}T}\frac{{Đ}_{i,p}}{x_{p}}\frac{d\mu_{i}}{dz}$$

where *N_i_* is the molecular flux of component *i*, *ρ_i_* is the number density of species *i* sorbed in the membrane, *Ð_i,p_* is the Maxwell–Stefan diffusivity of *i* in polymer *p*, *x_p_* is the polymer mole fraction in the membrane, *k_B_* is Boltzmann constant, *T* is temperature in Kelvin, and *μ_i_* is chemical potential of *i*. As written, Equation (1) is formulated for a flux of units (molecules/area/time). The Maxwell–Stefan diffusivities were extracted from the pure liquid hydraulic permeation data of Mathias et al. [8] via data fitting to Equation (1).

Recently, Thompson et al. [3] measured the membrane-based separation of a nine-component mixture (Table 1) at a feed composition of $x_{j}^{f}$. The separation coefficient *R_j_* is defined as the ratio of permeate mole fraction $x_{j}^{p}\;$to the feed or retentate mole fraction $x_{j}^{f}\;$(these experiments were conducted at low stage cuts), see Equation (2) below.

(2) $$R_{j}=\frac{x_{j}^{p}}{x_{j}^{f}}$$

**Figure 3:**
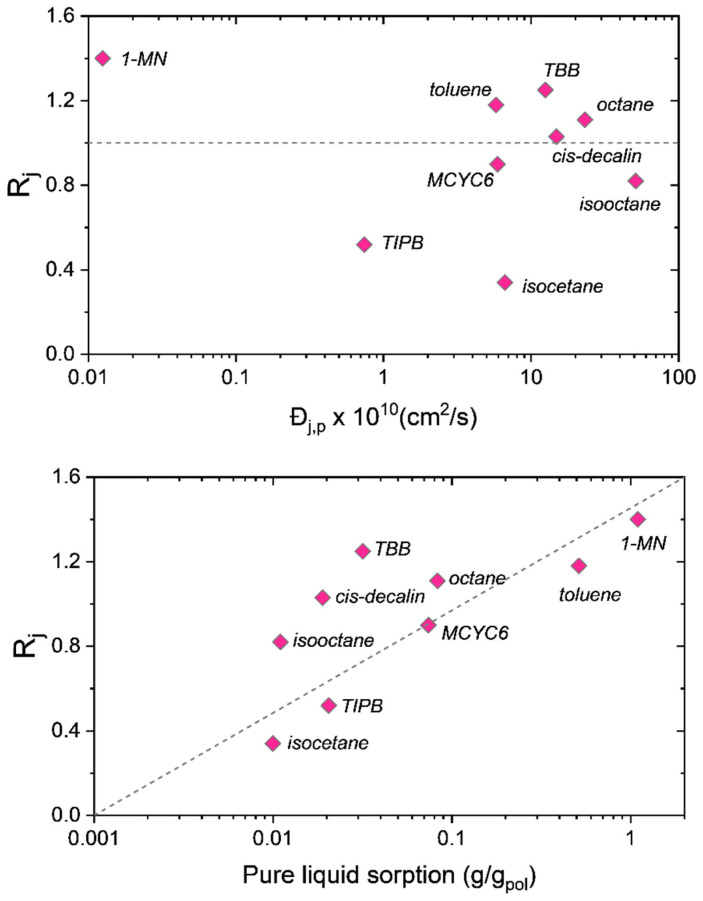
Separation coefficients of the nine-component mixture in the SBAD-1 membrane [3] versus Maxwell–Stefan diffusivities [9] (**top**) and sorption data from pure component liquids [8] (**bottom**). The membrane separation was done at a temperature of 22 °C and trans-membrane pressure 40 bar. Dashed lines are included as visual guides. MCYC6 = methylcyclohexane, TBB = tert-butylbenzene, TIPB = 1,3,5-triisopropylbenzene, 1-MN = 1-methylnaphthalene.

The top panel of Figure 3 plots the separation coefficients of the nine-component mixture versus the pure liquid diffusivities in the membrane, while the bottom panel plots the separation coefficients versus the pure liquid solubility (sorption) in the membrane. The top panel of Figure 3 shows no functional relationship between the separation coefficients and the pure component diffusivities. In fact, the species with the smallest diffusivity—1-MN, being nearly two orders of magnitude smaller—is the most purified in the permeate with an *R_j_* of 1.4. On the other hand, there is a clear functional dependence of *R_j_* on the pure liquid solubility. We note here that these diffusivities are on a molar basis, not a volumetric basis. We prefer the molar basis as it allows for a clear interpretation of the diffusivities, whereas the volumetric diffusivities commonly employed in polymer-based Maxwell–Stefan formulations require knowledge of solvent-averaged molecular weights of the membrane system [11].

Instead of acting as a semi-rigid material with selective molecule-by-molecule diffusion (as in the case of diffusion-selective gas separation), the polymer membrane in the organic liquid separation can instead be approximated as an extracting phase (e.g., liquid–solid–liquid extraction). To capitalize on this observation, Marshall et al. [9] proposed the following simple extension of Equation (1) to multi-component fluids containing *n* species,

(3) $$N_{i}=-\frac{\rho_{i}}{k_{B}T}\frac{{Đ}_{avg}}{x_{p}}\frac{d\mu_{i}}{dz}$$

where *Ð_avg_* is the average diffusivity of guest species in the membrane evaluated through the relation

(4) $${Đ}_{avg}=\sum_{i=1}^{n}{\hat{x}}_{i}^{m}{Đ}_{i,p}$$

where ${\hat{x}}_{i}^{m}$ is the mole fraction of *i* in the membrane on a guest species basis

(5) $${\hat{x}}_{i}^{m}=\frac{\rho_{i}}{\sum_{j=1}^{n}\rho_{j}}$$

Equation (3) assumes that the diffusion in the membrane is fully mutualized. When combined with an appropriate thermodynamic model to calculate the solubility and chemical potential gradients, Equation (3) was shown to accurately represent the separation of liquid mixtures in several glassy polymer membranes [9].

A consequence of Equation (3) is that the permeate mole fraction is independent of the diffusivities.

(6) $$x_{i}^{p}=\frac{x_{i}^{m}(z=0){\left(\frac{\partial \mu_{i}}{\partial z}\right)}_{z=0}}{\sum_{j=1}^{n}x_{j}^{m}(z=0){\left(\frac{\partial \mu_{j}}{\partial z}\right)}_{z=0}}\approx \frac{x_{i}^{m}\left(z=0\right)\left(\mu_{i}^{p}-\mu_{i}^{r}\right)}{\sum_{j=1}^{n}x_{j}^{m}\left(z=0\right)\left(\mu_{j}^{p}-\mu_{j}^{r}\right)}$$

where *z* = 0 is the axial location of the membrane–retentate boundary. In the second step of Equation (6), it is assumed the chemical potential varies linearly across the membrane (constant gradient approximation) [9]. $\mu_{i}^{p}$ is the chemical potential of *i* in the permeate and $\mu_{i}^{r}$ is the chemical potential in the retentate. The fraction $x_{i}^{m}\left(z=0\right)$ is the mole fraction of component *i* in the membrane at the retentate boundary.

Equation (6) provides a set of *n* equations that are solved iteratively to obtain the set of mole fractions $x_{i}^{p}$. Once this numerical solution has been obtained, the component flux of each species is easily calculated as follows (within the constant gradient approximation, ℓ in the denominator is the membrane thickness),

(7) $$N_{i}=x_{i}^{p}N_{total}$$

(8) $$N_{total}=\sum_{i=1}^{n}N_{i}=-\frac{{Đ}_{avg}}{k_{B}Tx_{p}\ell }\sum_{i=1}^{n}\rho_{i}\left(\mu_{i}^{p}-\mu_{i}^{r}\right)$$

Using Equation (6) it was demonstrated [9] that, when combined with the appropriate thermodynamic model, accurate predictions of the permeate compositions in SBAD-1 membrane-based separations of liquids could be made. Specifically, it was demonstrated that Equation (6) accurately predicted the separation highlighted in Figure 3, as well as the separation of a light shale crude oil on this same SBAD-1 membrane. 

A question arises from this prior work: was this good agreement a coincidence? Can the model accurately predict the temperature and pressure dependence of *R_j_* without further tuning? We will explore this question in the current work. Of course, if it is assumed that Equation (6) is reasonably correct, then the accurate prediction of the temperature and pressure dependence of the membrane selectivity is necessarily a test of the underlying thermodynamic model. As in our previous application, we employed the dry glass reference perturbation theory [12] (DGRPT) as our base thermodynamic model to calculate the solubility and chemical potential.

In our previous work [9], it was demonstrated that when Equation (6) is combined with DGRPT [12], the separation of the nine-component liquid mixture can be accurately predicted using only pure component vapor sorption isotherms at room temperature to parameterize the model. Furthermore, this same approach was applied to predict the SBAD-1 membrane-based separation of a light shale crude, and it accurately predicted the distillation curve of the permeate using a petroleum fraction representation based on the boiling point. While being useful for predicting the distillation properties, this approach did not allow for the prediction of the partitioning of molecular classes. In this work we represent the light shale crude using a detailed compositional model based on structure-oriented lumping [13], which allows for the prediction of the effect of molecular structures on enrichment in the permeate. We then demonstrated that the model predictions of the molecular class based separation are consistent with experimental data. As far as we are aware, this is the first example of such a model prediction and validation in the literature.

## 2. Theory and Model Development: Dry Glass Reference Perturbation Theory

As illustrated in Figure 1, glassy polymer membranes are exceptionally challenging to model due to the fact that their properties may vary substantially depending on the feed composition. Consider the case of a binary liquid mixture containing a non-dilating “C1” and a dilating “C2” species. In pure C1, the dilation of the membrane will be limited, and guest transport through the glassy polymer will be somewhat similar to gas transport mechanisms. While in pure C2, the membrane will be highly swollen, behaving more like an equilibrium liquid polymer. In this regime, equilibrium theories such as Flory–Huggins or statistical associating fluid theory (SAFT) [14] may be used. What happens for intermediate compositions? At what composition does the polymer transit from solid like to liquid like? Is it a smooth transition?

To answer these questions, an approach is needed which can bridge these two extremes. The non-equilibrium thermodynamics of glassy polymers [15,16,17,18,19] (NETGP) of Sarti and Doghieri was designed to address this gap. NETGP maps the non-equilibrium solubility in glassy polymers onto equilibrium equations of state.

Consider a fluid mixture composed of *n* species at temperature *T* and pressure *P*, in contact with a glassy polymer. The equilibrium relations for the *n* sorbing species are similar to the equilibrium case,

(9) $$\mu_{i}^{f}\left(T,P,\{x_{j}\}\right)=\mu_{i}^{poly}\left(T,\rho_{g},\{\rho_{j}\}\right)\;i,j=1-n$$

where $\mu_{i}^{f}\left(T,P,\left\{x_{j}\right\}\right)$ is the chemical potential of *i* in the fluid phase of mole fraction $\left\{x_{j}\right\}$. Since the fluid phase is considered an equilibrium fluid, the chemical potential is described as functions of *T*, *P* and $\left\{x_{j}\right\}$. Here, $\mu_{i}^{poly}\left(T,\rho_{g},\left\{\rho_{j}\right\}\right)$ is the chemical potential of *i* in the glassy polymer phase. It is specified as functions of *T*, the set of guest species number densities $\left\{\rho_{j}\right\}$, and the number density of the glassy polymer *ρ_g_*. In effect, *ρ_g_* has replaced *P* as a specification to the chemical potential. This is necessary in glassy polymers due to the fact that the thermodynamics pressure *P*, is not defined in a glassy polymer [9]. Both chemical potentials in the fluid phase and the polymer phase, Equation (9), are evaluated with equilibrium-density-explicit equations of state.

As it is assumed that the composition of the bulk fluid phase at the feed side is known, there are *n* + 1 unknown variables corresponding to all species densities in the polymer phase. However, there are only *n* equations provided by Equation (9). The additional relation in NETGP is obtained by treating the polymer density as an order parameter that must be specified. That is, the polymer density *ρ_g_* in the presence of sorbed guest species is not calculated self-consistently in the theory.

For non-swelling polymers, one can simply set $\rho_{g}=\rho_{g}^{o}$, where $\rho_{g}^{o}$ is the measured density of the solvent-free glassy polymer. We refer to this density as the “dry glass density”. For vapors/solvents that swell the polymer, setting $\rho_{g}=\rho_{g}^{o}$ gives poor results [20]. To compensate, the following correlation is often used:

(10) $$\frac{\rho_{g}}{\rho_{g}^{o}}=1-\sum_{i=1}^{n}k_{s,i}f_{i}$$

where *f_i_* is the component fugacity (equal to the partial pressure in ideal gases), and *k_s,i_* is an empirical coefficient which is adjusted to sorption data. Equations (9) and (10), when combined with a density explicit equilibrium equation of state, complete the NETGP approach.

NETGP has been highly successful at describing gas sorption [15,17,18,19] over a wide range of conditions. However, this formulation of NETGP is not well suited to describe sorption from complex liquid mixtures. First, the fugacity dependence of swelling is not necessarily linear. Second, Equation (10) does not account for competitive sorption effects. One can assume alternative non-linear fugacity dependence in Equation (10). However, it is difficult to utilize Equation (10) when different species may have swelling profiles with a different functional dependence on fugacity. While Equation (10) is sufficient to describe multi-component sorption from gases, it lacks the theoretical rigor to be applied for complex liquid mixtures.

Recently Marshall et al. [12] proposed an alternative closure relation to NETGP called dry glass reference perturbation theory (DGRPT). DGRPT develops a closure relation for the non-equilibrium polymer chemical potential by treating guest species sorption as the perturbation to a dry polymer reference state.

(11) $$\begin{array}{l} \mu_{p}^{poly}\left(T,\rho_{g},\{\rho_{k}\}\right)=\mu_{p}^{poly}\left(T,\rho_{g}^{o}\right)+\sum_{i=1}^{n}{\frac{\partial \mu_{p}^{poly}\left(T,\rho_{g},\{\rho_{k}\}\right)}{\partial \rho_{i}}|}_{\{\rho_{k}\}=0}\rho_{i}\\ \;\;\;\;\;\;\;+\frac{1}{2}\sum_{j=1}^{n}\sum_{i=1}^{n}{\frac{\partial^{2}\;\mu_{p}^{poly}\left(T,\rho_{g},\{\rho_{k}\}\right)}{\partial \rho_{j}^{}\partial \rho_{i}^{}}|}_{\{\rho_{k}\}=0}\rho_{i}^{}\rho_{j}^{}+\cdots \end{array}$$

$\mu_{p}^{poly}\left(T,\rho_{g},\left\{\rho_{k}\right\}\right)\;$ is the chemical potential of the polymer (subscript *p* refers to the polymer, and superscript *poly* to the polymer phase) as a function of the polymer density (*ρ_g_*) in the presence of the set of guest species densities (*ρ_k_*) and temperature *T*. The term $\mu_{p}^{poly}\left(T,\rho_{g}^{o}\right)$ represents the dry reference polymer chemical potential in the absence of sorbed species, i.e., $\left\{\rho_{k}\right\}$ = 0. It is assumed that the dry glass density is known. Finally, the derivatives in the expansion are evaluated in the limit of infinite dilution of guest species.

Equations (9) and (11) summarize the DGRPT approach. It is an alternative realization of NETGP, in which the theoretical closure Equation (11) is used in place of the empirical closure Equation (10). The major benefit of Equation (11) over Equation (10) is in its application to dilating mixtures. Equation (11) provides a closed framework without the need to make additional assumptions. This allows for applications to complex liquid mixtures over a wide range of conditions.

In the applications considered thus far [9,12], the accuracy has been sufficient when truncating Equation (11) at first order

(12) $$\mu_{p}^{poly}\left(T,\rho_{g},\{\rho_{k}\}\right)\approx \mu_{p}^{poly}\left(\rho_{g}^{o},T\right)+\sum_{i=1}^{n}{\frac{\partial \mu_{p}^{poly}\left(T,\rho_{g},\{\rho_{k}\}\right)}{\partial \rho_{i}}|}_{\{\rho_{k}\}=0}\rho_{i}$$

The dry glass density $\rho_{g}^{o}$ has a rheological dependence on the temperature and pressure. This dependence is accounted for with the following relation [9],

(13) $$\frac{\rho_{g}^{o}}{\rho_{g}^{oo}}=1+\frac{P}{\gamma }-\alpha \left(T-T_{o}\right)$$

$\rho_{g}^{oo}\;$is the dry glass density at the temperature *T_o_* and atmospheric pressure. The dry polymer modulus *γ* and thermal expansion coefficient α are dry pure polymer properties and are independent of any sorbed guest species. For bulk phase behavior calculations, *γ* is best interpreted as a bulk modulus, while for membrane operations, *γ* is interpreted as a dry Young’s modulus due to the uniaxial stress. Note, the actual modulus of the solvent swollen polymer is different from *γ*, and is calculated self-consistently in DGRPT.

Finally, an equilibrium equation of state for the evaluation of all chemical potentials must be specified. As in previous applications of DGRPT [9,12], we employed the simplified [21] polar [22] PC-SAFT [23] equation of state. In polar PC-SAFT, molecules are treated as chains of tangentially bonded spheres. The chain length *m*, sphere diameter *σ*, and sphere–sphere interactive energy *ε* are parameters in the model. In addition, polar species are further described by the polar strength *α_p_* = *mx_pol_μ*^2^, which provides the magnitude of polar attractions. Here, *x_pol_* is the fraction of segments in a molecule which are polar, and *μ* is the dipole moment of a polar segment.

PC-SAFT parameters for fluid phase species are fit to pure component vapor pressures and liquid densities [23]. For glassy polymers, equilibrium phase behavior does not exist to regress the *m*, *σ*, *ε* of the polymer (*α_p_* is set by the polymer molecular structure). Hence, these parameters are adjusted to the pure vapor sorption data of at least two vapor species with the polymer. In this work, we employed polymer parameters fit [12] in this way to SBAD-1. Toluene and heptane pure vapor sorption data at 25 °C were employed to extract [12] the polymer *m*, *σ*, *ε*.

For all remaining fluid phase species, if sorption data of a given molecule with the polymer exist, a binary interaction parameter *k_ij_* between the fluid phase species and the polymer can be adjusted. The binary interaction parameter is used in the combining rule for the cross species *ε_ij_* in terms of the pure component *ε*

(14) $$\varepsilon_{ij}=\sqrt{\varepsilon_{ii}\varepsilon_{jj}}\left(1-k_{ij}\right)$$

The pure component PC-SAFT parameters of SBAD-1 and fluid phase species are given in Table 2, while the binary interaction parameters between the fluid phase species and the polymer are given in Table 3. Table 3 also shows the fraction of aromatic carbon (*f_a_*), fraction of saturated carbon (*f_sat_* = 1 − *f_a_*), and the fraction of carbon which is alkane branches (*f_br_*) in these nine molecules, which will be applied for building correlations between them and the interaction parameters, to be discussed later.

The dry modulus γ does not have an effect for the low pressure vapor isotherms. However, for elevated pressures in liquid membrane separations, γ becomes important. As in the previous publication [9], we used *γ* = 0.7 GPa. Further, since the dry thermal expansion coefficient of SBAD-1 is unknown, we simply set *α* = 0. The dry glass density of SBAD-1 is $\rho_{g}^{oo}$ = 1.052 g/cc [3].

## 3. Results and Discussion:

### 3.1. Nine Component Mixture

We applied DGRPT in conjunction with Equation (6) to predict the membrane-based separation of a nine-component hydrocarbon mixture over a wide range of temperatures and pressures. DGRPT was used to calculate the guest species mole fraction $x_{i}^{m}$ within the polymer which is in equilibrium with the retentate. All polymer pure component PC-SAFT parameters (*m*, *σ*, *ε*) were fit to vapor phase toluene and n-heptane sorption data at 25 °C. Without any further adjustment, we applied the model to predict the membrane-based separation data using SBAD-1 measured by Thompson et al. [3]. The feed composition of the nine-component mixture is given in Table 4.

**Figure 4:**
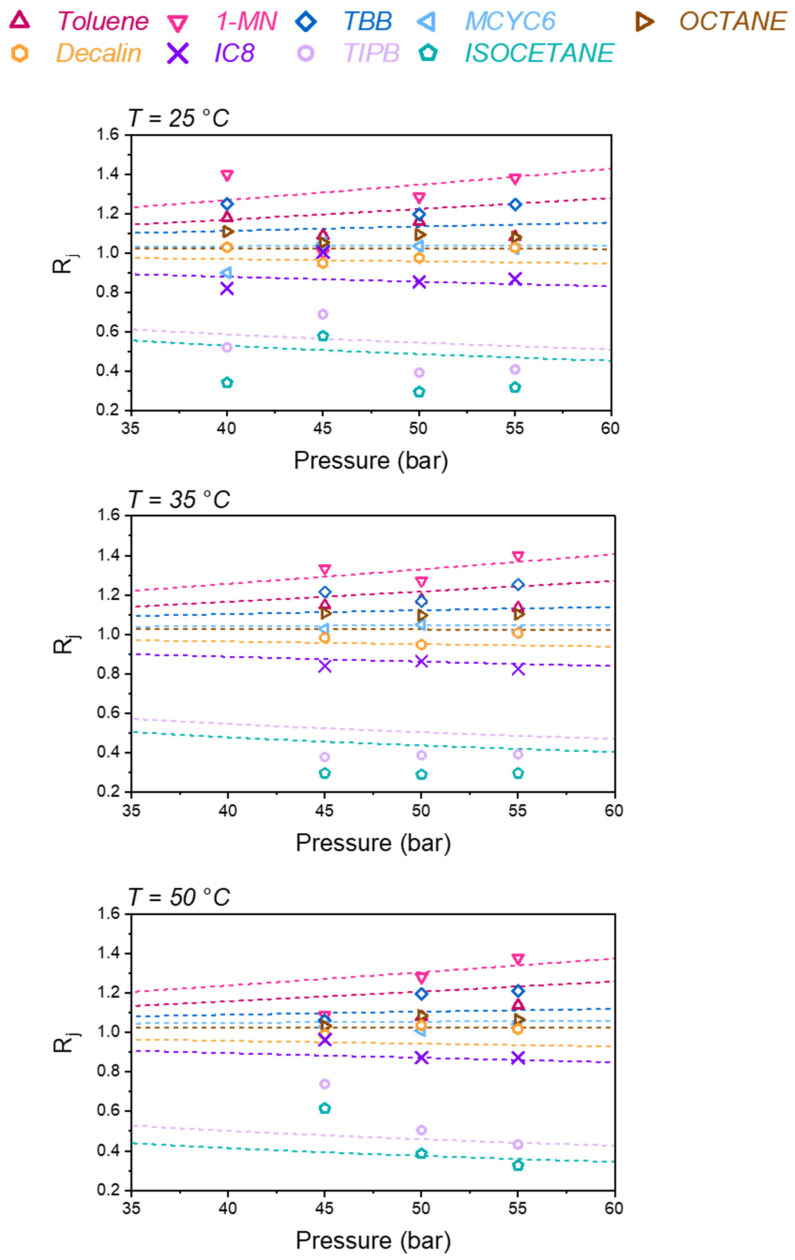
Separation coefficients (Equation (2)) for the SBAD-1 membrane separation of a complex liquid feed composition listed in Table 4. Symbols are experimental data [3] and curves are predictions using Equation (6). In the top panel, the 40 bar experimental data are for *T* = 22 °C and the remaining data are at 25 °C.

Figure 4 compares model predictions using Equation (6) to data [3] for the separation coefficients (Equation (2)) of the SBAD-1 membrane separation of this mixture. Pressure refers to the retentate pressure, while the permeate pressure is atmospheric. Overall, the agreement between model and experiment is quite good considering the complexity of this system.

As expected from our previous work [9], the model does a good job predicting the overall order of the separation at 25 °C and 40 bar. The model accurately predicts that 1-methylnaphthalene is the most purified in the permeate stream. These results demonstrate the accuracy of the model for the temperature and pressure dependence of the separation. It should be noted that the data at 45 bar may be of a lower accuracy. To demonstrate this, we included data at 40 bar and 22 °C (top panel) which were measured [3] on a separate unit. As can be seen, the model is in better agreement with the data at 40 bar, than at 45 bar. We believe the measured data at 45 bar are in error at both 25 °C and 50 °C.

Both the model and data suggest that increasing the pressure increases the purity of the species with *R_j_* > 1, and decreases the purity of species with *R_j_* < 1 in the permeate. This is consistent with typical osmotic separations in which higher pressures incrementally compensate against osmotic resistances to permeation.

**Figure 5:**
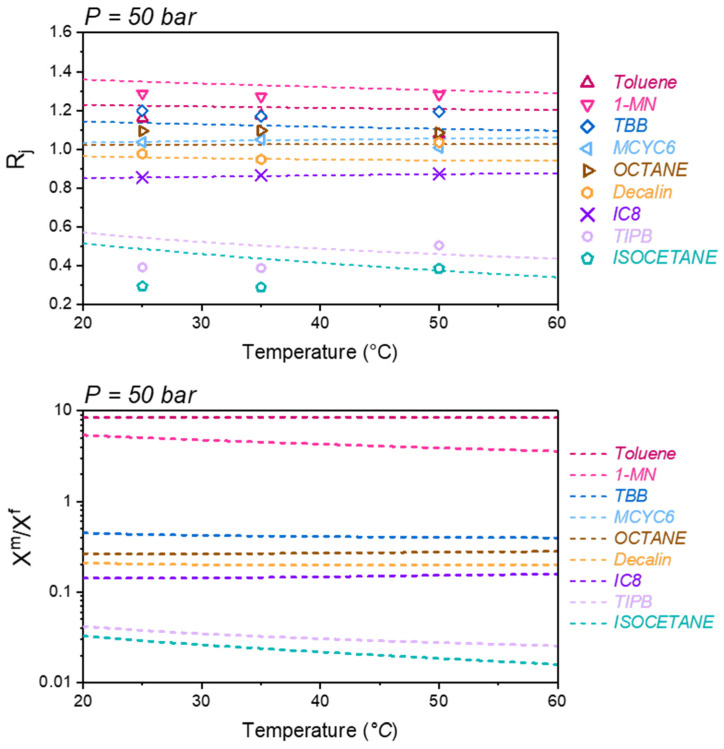
Top: Separation coefficients (Equation (2)) for the SBAD-1 membrane separation of a complex liquid feed composition listed in Table 4, at a fixed feed pressure of 50 bar. Symbols are experimental data [3] and curves are predictions using Equation (6). (**Bottom**): Same as (**top**) except plotting model predictions of the ratio of mole fraction in membrane at the feed interface to the feed.

The effect of temperature is more subtle, and is best observed at fixed pressure. Figure 5 compares the model and data for the separation coefficients as a function of temperature, while at a fixed feed pressure of 50 bar. Overall, the model predicts that only the most purified (1-MN) and rejected (isocetane, TIPB) components have a significant temperature dependence in the separation coefficient. The model predicts that *R_j_* decreases for the purified species 1-MN, toluene, and TBB. The small magnitude of this decrease appears to be within the uncertainty of experimental data.

The model predicts that *R_j_* decreases for the rejected species isocetane and TIPB, while the data appear to show the opposite trend of increasing *R_j_* with increasing temperature. This disagreement may be due to the data uncertainty, considering that both species are dilute in the feed, and small errors in composition measurements could give rise to this apparent discrepancy. The discrepancy may be also due to the model error.

In the dry glass reference density given by Equation (13), we have assumed the dry polymer thermal expansion coefficient of SBAD-1 as zero. If instead, we assume the dry polymer thermal expansion coefficient is equal to that of Matrimid (1.89 × 10^−4^ K^−1^), the model predictions are not significantly affected. To bring the model predicted temperature dependence in line with experiment, we must use α = 1.5 × 10^−3^ K^−1^. However, this value of thermal expansion coefficient is too large to be considered for a dry glassy polymer, which was not applied in this work.

In addition, the binary interaction parameters used in the model, as seen in Table 3, do not have any temperature dependence. Even in the application of PC-SAFT to rubbery systems [25], binary interaction parameters for solvent–polymer pairs are often temperature dependent. Given the uncertainties in the data, our goal is not to adjust the model to best reproduce the data in Figure 4 and Figure 5. Instead, these results demonstrate that Equation (6) combined with DGRPT can make good qualitative predictions of the temperature and pressure dependence of the separation of this complex liquid mixture, using only vapor sorption data at 25 °C to parameterize the model.

The bottom panel of Figure 5 plots model predictions of the ratio of mole fraction in the membrane $\;x_{j}^{m}$ to the feed mole fraction, $x_{j}^{f}$. As can be seen, toluene and 1-MN are concentrated within the membrane, $x_{j}^{m}>x_{j}^{f},$ while the remaining species are depleted $x_{j}^{m}<$ $x_{j}^{f}$. When comparing the temperature dependence of $x_{j}^{m}$ to that of *R_j_*, it is clear that the model predicted that the temperature dependence of *R_j_* is dominated by the temperature dependence of $x_{j}^{m}$.

### 3.2. Compositional Modelling of a Light Shale Crude Oil

Thompson et al. [3] measured the separation of a light shale crude oil using an SBAD-1 membrane. In our previous work [9], we demonstrated how DGRPT could be combined with Equation (6) to predict the membrane separation of this light shale crude. The crude oil composition was determined using petroleum pseudo-components which are defined on the basis of boiling point temperature. While this approach was able to predict the distillation properties of the permeate, sufficient compositional information was not available to predict how certain classes of molecules were enriched in the permeate.

To make detailed compositional predictions, a detailed composition of the crude oil is needed. This detailed compositional model is provided by the structure-oriented lumping (SOL) approach of Quann and Jaffe [13]. SOL is a group representation of petroleum molecules, which is well suited for creating reaction networks. Models of composition based on SOL are constructed through large scale experimental programs and extensive modeling efforts to derive the concentration of thousands of hydrocarbon molecules within the crude oil. The details of this procedure are ExxonMobil proprietary; however, they are not needed in the following discussion.

We now apply Equation (6) with DGRPT to the SBAD-1 membrane separation of the light shale crude using a SOL-based model of composition to represent the feed. To apply DGRPT to this complex mixture, we must generate pure component PC-SAFT parameters *m*, *σ*, *ε*, *α_p_* for each petroleum species. For this, we used the approach of Marshall et al. [26,27] which accurately generates PC-SAFT parameters on the basis of pure component boiling point temperature (*T_b_*), specific gravity (*SG*), molecular weight (*MW*) and the fraction of carbon which is aromatic (*f_a_*). The molecular weight and *f_a_* are defined in each SOL-lumped species, and specific correlations for *T_b_* and *SG* are from ExxonMobil proprietary models. Therefore, the SOL model of composition provided all the required information to derive the PC-SAFT pure component parameters for the crude oil.

In addition to pure component parameters, binary interaction parameters are needed between the hydrocarbon species and SBAD-1. To accomplish this, a correlation must be developed which depends on the structure of the hydrocarbons. As can be seen in Table 3, aromatic species have smaller binary interaction parameters than saturated species. This is due to the stronger attraction with the aromatic polymer backbone. On the other hand, branched species have larger binary interaction parameters. This signifies a decrease in attraction between the hydrocarbon and the polymer. For this reason, we considered three descriptors: fraction of aromatic carbon (*f_a_*), fraction of saturated carbon (*f_sat_* = 1 − *f_a_*), and fraction of carbon that contains alkane branches (*f_br_*). These fractions are included in Table 3 for the nine hydrocarbons for which we have regressed binary interaction parameters with SBAD-1. We did not include the fraction of naphthenic carbon as there is not currently substantial evidence to suggest that paraffin versus naphthenic interactions with the polymer necessitate descriptors in the *k_ij_* model. This can be seen in Table 3, i.e., both methylcyclohexane and octane have *k_ij_* ~0.06. It should be noted that molecular weight dependence is built in theoretically in DGRPT through PC-SAFT.

We propose the following simple correlation for the binary interaction parameter between a hydrocarbon and the SBAD-1 polymer,

(15) $$k_{ij}=c_{a}f_{a}+c_{sat}\left(1-f_{a}\right)+c_{br}f_{br}$$

**Figure 6:**
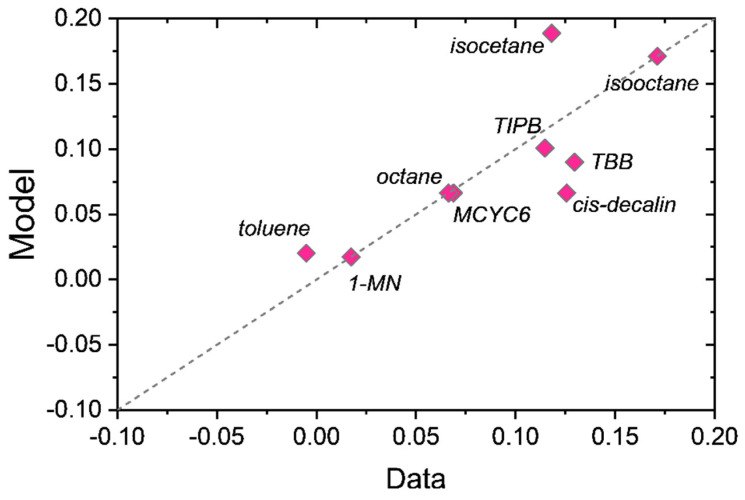
Parity plot of correlation predicted binary interaction parameters (Equation (15)) versus data values from Table 3.

The coefficients *c_a_*, *c_sat_*, and *c_br_* are fitted to the binary interaction parameters in Table 3. The coefficients are listed in Table 5. As shown in the parity plot Figure 6, Equation (15) gives a reasonable correlation of the binary interaction parameters.

**Figure 7:**
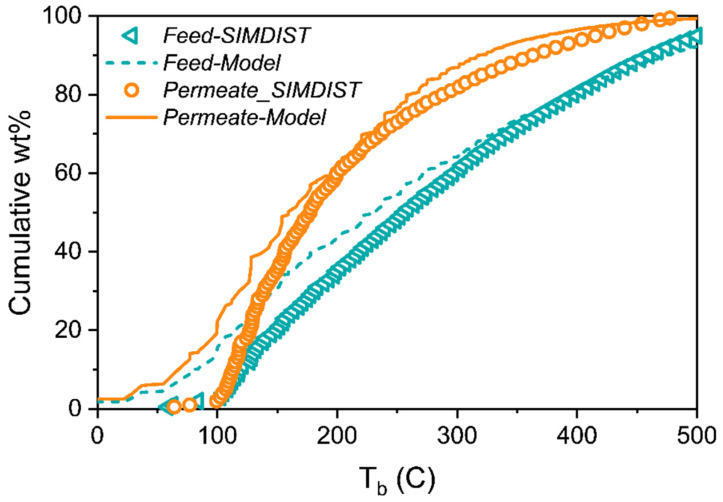
Comparison of the feed SIMDIST [3] to model of composition for a light shale crude oil, and prediction of DGRPT for the permeate SIMDIST [3] at 130 °C and 55 bar vs. measured values.

With the model now fully developed, we make predictions for the separation of a light shale crude at 55 bar and 130 °C. We compare the model’s prediction to the experimental data of Thompson et al. [3]. Figure 7 compares the model of composition to the feed simulated distillation (SIMDIST) data, as well as model predictions of the permeate SIMDIST to the measured data. It should be noted that there is a discrepancy in the low boiling point range between the model of composition and the feed SIMDIST. This is due to the loss of low-boiling species (vaporizing quickly during the sample handling) in the SIMDIST measurement. The same thing happened for the SIMDIST measurement of the permeate stream.

**Figure 8:**
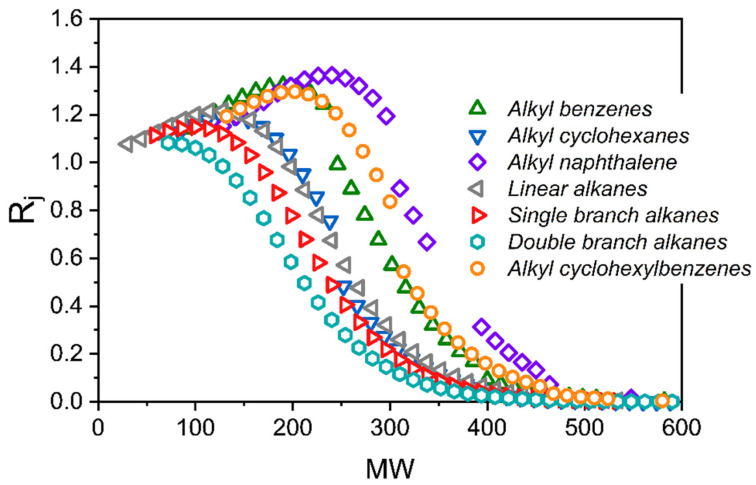
Model predicted separation coefficients versus molecular weight for several molecular classes at 130 °C and 55 bar.

SIMDIST curves illustrate the boiling point distribution of a petroleum stream, but do not convey molecular compositional details. In Figure 8 the separation coefficient, Equation (2), versus molecular weight is plotted for several molecular classes based on the model’s prediction.

A common feature of most molecular classes is that *R_j_* is close to 1 for small molecular weights, which initially increases with an increasing molecular weight going through a maximum and then decreases to ~0 at high molecular weights. The maximum is a result of an initial increase in solubility with increasing molecular weight, and then a subsequent decline in solubility with increasing molecular weight. Double branched alkanes are the only class that do not exhibit the increase in *R_j_* at low molecular weights, or the corresponding maximum.

**Figure 9:**
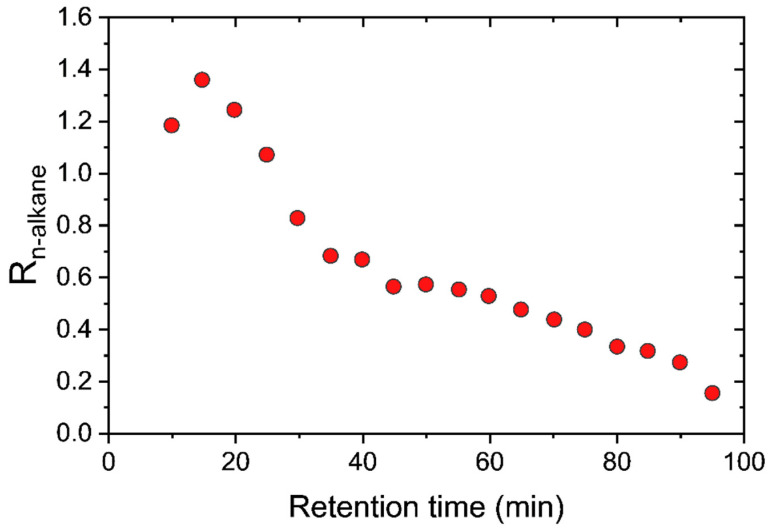
2D-GC measurements [3] of the n-alkane separation coefficients versus retention time in the light shale crude oil separation.

The maximum in *R_j_* is supported by the two-dimensional gas chromatography (2D-GC) results of Thompson et al. [3]. Figure 9 plots 2D-GC measurements of *R_j_* versus retention time for the n-alkane series in the light shale crude oil separation. Increasing the retention time corresponds to an increasing molecular weight. Thompson et al. [3] reported the retention time of n-octane (MW = 114.23 g/mol) to be ~20 min. In Figure 9, the retention time of 20 min is just to the right of the maximum, which occurs at ~15 min, corresponding to n-heptane. The model predicts (Figure 8) that n-nonane exhibits the maximum separation factor for the n-alkane series. This prediction is remarkably accurate (C_9_ vs. C_7_), given the fact that the alkane carbon numbers in the SOL input range from C_2_–C_70_. In addition, using 2D-GC, Thompson et al. measured the enrichment of n-alkanes in the permeate stream to be ~21%. This is in good agreement with the DGRPT model prediction of ~23%, as shown in Table 6.

Table 6 lists the model predictions of enrichment of each molecular class shown in Figure 8. The enrichment of molecular class *c*, *E_c_*, is calculated as

(16) $$E_{c}=\frac{w_{c,p}}{w_{c,f}}-1$$

where *w_c,p_* is the weight fraction of a full molecular class in the permeate, and *w_c,f_* is the weight fraction of a full molecular class in the feed. As can be seen, each class is enriched, except for the double branched alkanes, which are strongly rejected.

## 4. Conclusions

In this work we have further validated the use of a simplified membrane model, Equation (6) with DGRPT, as a versatile tool to predict the separation performance of glassy polymer membranes. We applied the model to the separation of complex liquid mixtures using SBAD-1 membranes. We demonstrated that the separation of the nine-component liquid mixture with this membrane is solubility driven. For this reason, it is possible to predict the permeate composition through Equation (6) without the use of diffusivities, although diffusivities are still needed to predict the fluxes.

The model accurately predicts the temperature and pressure dependence of the nine-component mixture separation. In addition, when combined with a light shale crude oil composition, the methodology predicts the structural dependence on permeate enrichment. The model predicts a non-monotonic molecular weight dependence within molecular classes. This prediction is consistent with the 2D-GC measurements of Thompson et al. [3].

The model input parameters are the pure polymer PC-SAFT parameters *m*, *σ*, *ε*, *α_p_* and the binary interaction parameters between the nine hydrocarbon species and the polymer. These parameters were determined solely from pure component vapor phase sorption measurements at 25 °C. However, the model was able to make robust predictions for the temperature and pressure dependence of complex liquid mixture separations, using only room temperature vapor sorption measurements to parameterize the model.

## Figures and Tables

**Table 1:** List of nine hydrocarbon components and their acronyms.

octane	*cis*-decalin
1-methylnaphthalene (1-MN)	isocetane
toluene	tert-butylbenzene (TBB)
Methylcyclohexane (MCYC6)	1,3,5-triisopropylbenzene (TIPB)
isooctane	

**Table 2:** Pure component polar PC-SAFT parameters for nine hydrocarbons and SBAD-1. The molecular weight of SBAD-1 is assumed to be *MW* = 100,000 g/mol. MCYC6 = methylcyclohexane, TBB = tert-butylbenzene, TIPB = 1,3,5-triisopropylbenzene, 1-MN = 1-methylnaphthalene.

Component	*m*	*σ*(Å)	*ε/k_b_*(K)	*α_p_*(D)	Ref.
1-MN	3.163	3.998	354.70	3.6	[12]
MCYC6	2.675	3.989	281.63	0	[12]
TIPB	5.471	3.922	255.83	2.16	[12]
TBB	3.459	3.953	284.62	2.16	[12]
isooctane	3.144	4.091	249.63	0	[12]
isocetane	5.016	4.301	266.58	0	[12]
octane	3.841	3.819	242.13	0	[12]
toluene	2.612	3.814	293.33	2.16	[24]
SBAD-1	0.0397 *MW*	2.963	124.13	0.028 *MW*	[12]

**Table 3:** Binary interaction parameters [12] between hydrocarbons and SBAD-1. Fraction of aromatic carbon (*f_a_*), fraction of saturated carbon (*f_sat_* = 1 − *f_a_*), and fraction of carbon which is alkane branches (*f_br_*). MCYC6 = methylcyclohexane, TBB = tert-butylbenzene, TIPB = 1,3,5-triisopropylbenzene, 1-MN = 1-methylnaphthalene.

Component	*k_ij_*	*f_a_*	*f_sat_*	*f_br_*
octane	0.0663	0	1	0
1-MN	0.0174	0.91	0.09	0
toluene	−0.0051	0.857	0.143	0
MCYC6	0.0688	0	1	0
isooctane	0.1712	0	1	0.375
*cis*-decalin	0.1256	0	1	0
isocetane	0.1181	0	1	0.437
TBB	0.1296	0.6	0.4	0.2
TIPB	0.1147	0.4	0.6	0.2

**Table 4:** Nine-component mixture for SBAD-1 membrane separation from Thompson et al. [3].

Component	$x_{j}^{f}$
octane	0.22
1-MN	0.02
toluene	0.171
MCYC6	0.281
isooctane	0.15
*cis*-decalin	0.11
isocetane	0.013
TBB	0.022
TIPB	0.016

**Table 5:** Coefficients for the general binary interaction parameter model between hydrocarbons and SBAD-1.

*c_a_*	*c_sat_*	*c_br_*
0.01252	0.0663	0.2797

**Table 6:** Model predictions of molecular class enrichment in the permeate (Equation (16)) at 130 °C and 55 bar.

Class	% Enrichment
Linear alkanes	23.1
Single branch alkanes	19.0
Double branch alkanes	−43.4
Alkyl cyclohexanes	11.9
Alkyl benzenes	35.3
Alkyl cyclohexylbenzenes	0.3
Alkyl naphthalenes	39.1
